# Crosstalk among the proteome, lysine phosphorylation, and acetylation in romidepsin-treated colon cancer cells

**DOI:** 10.18632/oncotarget.10840

**Published:** 2016-07-26

**Authors:** Tian-Yun Wang, Yu-Rong Chai, Yan-Long Jia, Jian-Hui Gao, Xiao-Jun Peng, Hua-Feng Han

**Affiliations:** ^1^ Department of Biochemistry and Molecular Biology, Xinxiang Medical University, Henan, 453003, China; ^2^ Henan Collaborative Innovation Canter of Molecular Diagnosis and Laboratory Medicine, Xinxiang,Henan, 453003, China; ^3^ Department of Histology and Embryology, School of Basic Medical Sciences, Zhengzhou University, Zhengzhou, Henan, 450001, China; ^4^ Pharmacy Collage, Xinxiang Medical University, Xinxiang, 453003, Henan, China; ^5^ Jingjie PTM BioLab (Hangzhou) Co. Ltd, Hangzhou, 310018, China

**Keywords:** romidepsin, colon cancer, histone lysine-acetylation, proteome, histone lysine-phosphorylation

## Abstract

Romidepsin (FK228) is one of the most promising histone-deacetylase inhibitors due to its potent antitumor activity, and has been used as a practical option for cancer therapy. However, FK228-induced changes in protein modifications and the crosstalk between different modifications has not been reported. To better understand the underlying mechanisms of FK228-related cancer therapy, we investigated the acetylome, phosphorylation, and crosstalk between modification datasets in colon cancer cells treated with FK228 by using stable-isotope labeling with amino acids in cell culture and affinity enrichment, followed by high-resolution liquid chromatography tandem mass spectrometry analysis. In total, 2728 protein groups, 1175 lysine-acetylation sites, and 4119 lysine-phosphorylation sites were quantified. When the quantification ratio thresholds were set to > 2.0 and < 0.5, respectively, a total of 115 and 38 lysine-acetylation sites in 85 and 32 proteins were quantified as increased and decreased targets, respectively, and 889 and 370 lysine-phosphorylation sites in 599 and 289 proteins were quantified as increased and decreased targets, respectively. Furthermore, we identified 274 proteins exhibiting both acetylation and phosphorylation modifications. These findings indicated possible involvement of these proteins in FK228-related treatment of colon cancer, and provided insight for further analysis of their biological function.

## INTRODUCTION

Aberrant transcriptional repression of genes regulating cell growth and differentiation is a hallmark of cancer [1]. Altered activation of histone deacetylases (HDACs) is a key mechanism underlying transcriptional repression in malignancies [2], and in solid tumors, including colon cancer and malignant melanoma, HDAC overexpression is believed to similarly contribute to oncogenesis [3]. Accordingly, the use of small compounds that inhibit HDAC activity has become a novel strategy for cancer treatment [4, 5]. HDAC inhibitors (HDACis) are able to restore the expression of aberrantly suppressed genes capable of inhibiting tumor cell growth and/or survival [6, 7]. Previously investigated HDACis include suberoylanilide hydroxamic acid (SAHA) [8, 9], depsipeptide (romidepsin), sodium phenylbutyrate [10, 11], and MS-275 [12, 13].

Romidepsin (FK228) is a novel HDACi that was approved by the United States Food and Drug Administration for treatment of cutaneous T cell lymphoma in 2009 and peripheral T cell lymphoma in 2011 [14, 15]. Its activities were also confirmed against other solid-tumor cancers, such as non-Hodgkin's lymphoma [16, 17], lung cancer [18, 19], breast cancer [20, 21], and colon cancer [22, 23]. FK228 exerts an antitumor effect through histone hyperacetylation and the subsequent transcription of genes that inhibit tumor growth [24, 25].

Although FK228 is already used in clinical trials, its mechanism of action remains largely undefined. Protein post-translational modifications (PTMs) function as highly versatile switches that regulate protein activity, concentration, subcellular localization, and maintain homeostasis. Chen et al. [26] performed a differential proteomic analysis to identify proteins associated with FK228-induced cytotoxicity in human lung cancer cells, identifying 27 proteins involved in signal transduction, transcription regulation, metabolism, and cytoskeletal organization. Wu et al. [27, 28] found that acetylation and ubiquitination sites changed in response to SAHA treatment, and that 55 common sites were capable of being both acetylated and ubiquitinated, with ubiquitination levels in 43 sites (78.2%) correlated with acetylation levels. Additionally, FK228-induced histone hyperacetylation in lung carcinoma cells was associated with increases in p21 and hypophosphorylated retinoblastoma protein expression [29].

To expand on these findings and reveal relationships between phosphorylation and the acetylome in response to FK228 treatment, we performed stable isotope labeling by amino acids (SILAC), affinity enrichment by antibodies, and high-resolution liquid chromatography tandem mass spectrometry (LC-MS/MS) to quantitatively compare the proteome, global phosphorylation levels, and the acetylome of HCT-8 and HCT-116 colon cancer cells before and after FK228 treatment. Additionally, the crosstalk between phosphorylation events and the acetylome was also analyzed in order to strengthen our understanding of FK228-dependent cancer therapy.

## RESULTS

### Cell cytotoxicity assay

**Figure 1:**
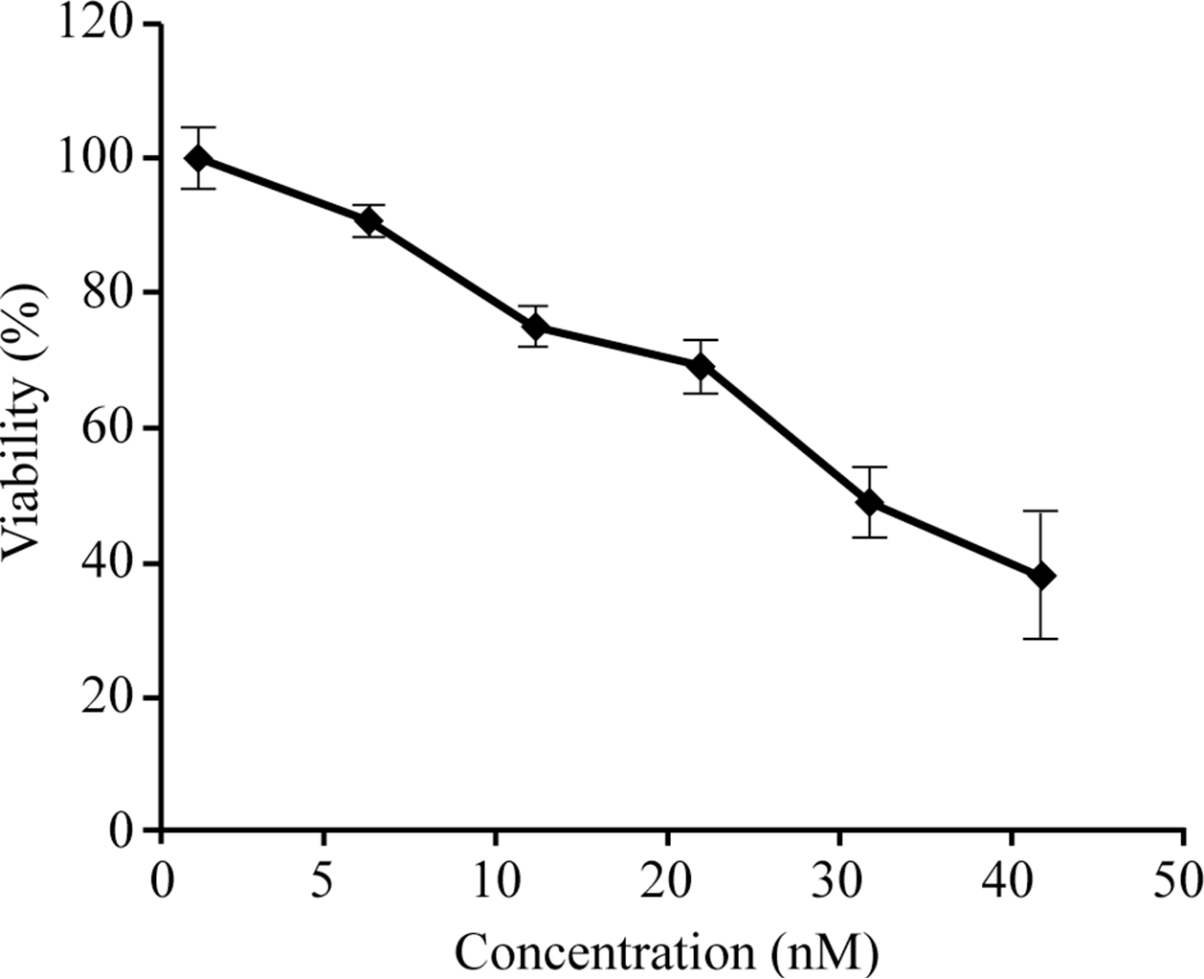
Establishment of the appropriate FK228 concentration in HCT-116 cells HCT-116 cells were cultured, and cell-proliferation and cytotoxicity assays were performed. The FK228 concentration resulting in 50% cell viability (IC_50_) was used as the working concentration.

Previous reports indicated that 50% of the HCT-8 cells were viable (IC_50_) at FK228 concentrations of 29.46 nM [30]; therefore, this dosage was used for the 18-h treatment period in all subsequent experiments. In this study, we performed cytotoxicity assays on HCT-116 cells using varying concentrations of FK228. Our results demonstrated a dose-dependent response, whereby the viability of cultured HCT-116 cells decreased in response to increasing FK228 concentrations (Figure 1). Notably, ~50% of the cells were viable (IC_50_) at a FK228 concentration of 26.86 nM, which was similar to the previous study [31]. Therefore, this dosage was used for the 18-h treatment period for HCT-116 cells in all of the subsequent experiments.

### Proteome profile changes following FK228 treatment

**Figure 2:**
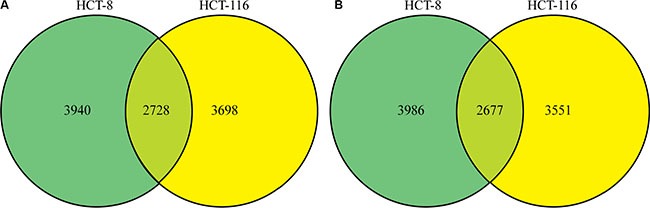
Changes in the proteome profile following FK228 treatment between HCT-8 and HCT-116 cells Quantitative lysine-acetylome and global-phosphorylation analyses were performed in HCT-8 and HCT-116 cells using SILAC and affinity enrichment, followed by high-resolution LC-MS/MS analysis. Total and differentially modified proteins and regulation patterns were identified. (**A**) Differentially regulated protein groups between the two cell lines. (**B**) Differential regulation patterns between the two cell lines.

Quantitative lysine-acetylome and global-phosphorylation analyses were performed in triplicate on HCT-8 and HCT-116 cell lines using SILAC and affinity enrichment, followed by high-resolution LC-MS/MS analysis. In total, 4910 protein groups were identified, with 4860 of them exhibiting differential regulation patterns between FK288-treated and -untreated cells, including 3940 and 3698 protein groups and 3986 and 3551 differential regulation patterns in HCT-8 and HCT-116 cells, respectively. We identified 2728 common protein groups and found that 2677 of them exhibited differential regulation patterns between FK288-treated and -untreated cells in the two cell lines (Figure 2A and 2B). After setting quantification ratios to > 2.0 and < 0.5 representing the increased- and decreased-regulation thresholds, respectively, among the 2677 common quantifiable proteins, 684 were classified as targets of increased regulation and 321 as targets of decreased regulation. Within these targets, 889 phosphorylation sites in 599 proteins were identified as exhibiting increased phosphorylation and 370 sites in 289 proteins were identified as exhibiting decreased phosphorylation, while 115 lysine-acetylation sites in 85 proteins were identified exhibiting increased acetylation and 38 sites in 32 proteins were identified as exhibiting decreased acetylation. These data are listed in Supplementary Table S1.

### Alterations of phosphorylation profiles in FK228-treated cells

Phosphorylation involves the addition of a phosphate (PO_4_^3−^) group to a protein or other organic molecule and is among the most important PTMs. Phosphorylation stimulates the active state of many enzymes, thereby altering their function. Additionally, phosphorylation is involved in a wide range of cellular processes, including cell signaling, immune-system modulation, and tumor suppression [32–34].

In this study, we combined SILAC and affinity enrichment, followed by high-resolution LC-MS/MS analysis, for quantitative phosphoproteomics analysis in HCT-8 and HCT-116 cells with or without FK228 treatment.

**Figure 3:**
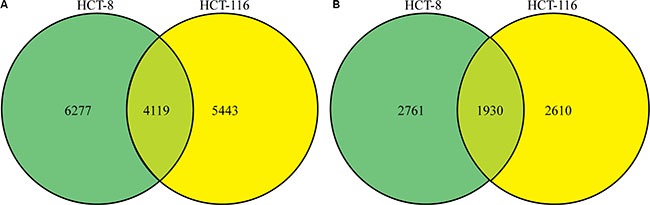
Phosphorylation profile changes following FK228 treatment of HCT-8 and HCT-116 cells SILAC, affinity enrichment, and high-resolution LC-MS/MS analysis was used for quantitative phosphoproteomics analysis of HCT-8 and HCT-116 cells following FK228 treatment. Total and differentially phosphorylated sites and phosphorylated proteins were assessed. (**A**) Sites exhibiting differential phosphorylation patterns in the two cell lines. (**B**) Differentially phosphorylated proteins between the two cell lines.

Altogether, 7601 phosphorylation sites in 3441 proteins were identified as differing between treated and untreated cells, resulting in quantification of 6178 sites in 2316 proteins. We found that 4119 common phosphorylation sites in 1930 proteins differed between treated and untreated HCT-8 and HCT-116 cell lines (Figure 3A and 3B). After setting quantification ratios to > 2.0 and < 0.5 as representing thresholds of increased and decreased phosphorylation events, respectively, 889 phosphorylation sites in 599 proteins were identified as targets exhibiting increased phosphorylation and 370 sites in 289 proteins were identified as targets exhibiting decreased phosphorylation. All data are listed in Supplementary Table S2.

### GO analysis and protein domains involved in phosphorylation

**Figure 4:**
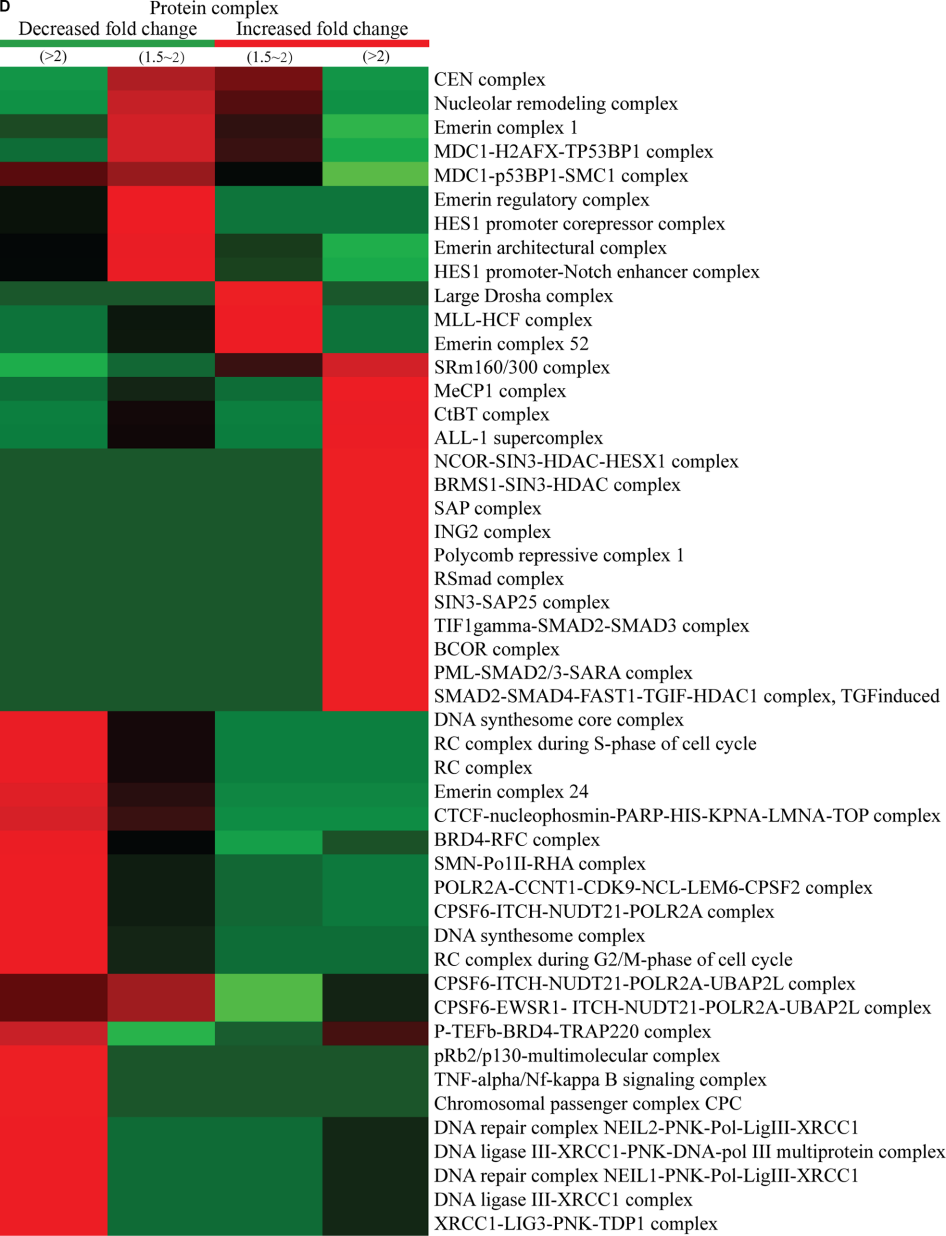
Functional enrichment-based clustering analysis for the quantified phosphorylated proteins According to the functional differences observed between increased and decreased proteins, GO-term association and enrichment analysis using the DAVID program (https://david.ncifcrf.gov/) were performed. (**A**) Biological process analysis. (**B**) Cellular component analysis. (**C**) Molecular function analysis. (**D**) Protein domain analysis.

According to the functional differences observed between increased and decreased phosphorylation events, we analyzed the proteins with quantified phosphorylation sites using GO enrichment-based clustering (Figure 4A−4C). Using the same threshold significance ratios, we performed these clustering analyses by dividing all proteins exhibiting statistically significant alterations into four quantiles (Q1−Q4) according to the L/H ratios (Q1: < 0.50, Q2: 0.50−0.67, Q3: 1.50−2.0, Q4: > 2.0) in order to determine the biological functions of the proteins exhibiting large (> 2.0 or < 0.50) or relatively small changes in ratios (1.5−2.0 or 0.50−0.67) in response to FK228 treatment.

The results of our analyses of the biological processes associated with phosphorylation are shown in Figure 4A. These results indicated that the proteins exhibiting decreased lysine phosphorylation in the Q1 quantile were highly enriched for cell division and cell cycle functions and regulation of transmembrane transport. Proteins with functions involving antigen processing and presentation, response to toxic substances, lipid localization, lipid transport, blood circulation, circulatory system processes, macromolecular methylation, and microtubule-based movement were segregated into the Q2 quantile. The proteins exhibiting increased lysine phosphorylation were found to be involved in many biological process, such as female gamete generation, protein localization to the adherens junction, and some response proteins, including those involved in responses to alcohol, drugs, hepatocyte growth-factor stimulus, and indole-3-methanol. There were some proteins involved in regulatory functions, such as regulation of epithelial cell differentiation, cellular-component size, and actin filament-based movement.

The cellular-component analysis revealed that proteins in Q1 and Q2 exhibiting decreased phosphorylation events were preferentially located at chromosomal regions, such as the contractile ring and kinetochore, as well as other areas, including the myelin sheath and postsynaptic regions. These proteins were classified as brush border and nuclear inner-membrane proteins (Figure 4B). In contrast, the proteins exhibiting increased phosphorylation events were constituents of nuclear and cell organelles, such as the nuclear inner membrane, nuclear euchromatin and heterochromatin, rDNA heterochromatin, and the mitochondrial membrane. Some proteins were involved in kinase complexes, such as the AMP-activated protein kinase complex and histone deacetylase complex.

We also analyzed the molecular functions associated with phosphorylation events (Figure 4C). The proteins exhibiting decreased phosphorylation were highly enriched in phosphorylation-related processes, including binding proteins, such as ribosomal small-subunit binding, ankyrin binding, Y-form DNA binding, damaged-DNA binding, and 1-phosphatidylinositol binding. Methyltransferase activity was also enriched in phosphorylation-related processes, such as lysine *N*-methyltransferase activity and histone methyltransferase activity. Moreover, other proteins were involved as structural constituents of muscle and channel-regulator activity. Furthermore, proteins exhibiting decreased phosphorylation levels were also involved in RNA transcription and translation, such as DNA-directed RNA polymerase activity, RNA polymerase activity, DNA helicase activity, DNA-directed DNA polymerase activity, Rab guanyl-nucleotide-exchange factor activity, and ATPase capacity. Proteins exhibiting increased phosphorylation levels were enriched in nucleic acid binding, receptor-regulator activity, NAD-dependent enzyme activity, hydrolase activity, and ligase activity.

Following protein-domain analysis, we observed that Q1 was enriched with proteins having ribosomal protein domains, domains containing a sterile alpha motif, homeodomain-like structures, LEM-like domains, a rabaptin-like structure, a SET domain, a post-SET domain, NOP domains, and thioredoxin domains. Furthermore, some disease-associated domains were also observed, including retinoblastoma-associated domains and LisH-dimerization motifs, whereas Q2 was enriched with proteins containing lambda repressor-like domains, ABC-transporter domains, and guanylate-kinase domains. In contrast, Q3 was enriched with proteins exhibiting increased phosphorylation and having Kelch-type beta-propeller domains and dehydrogenase domains, while Q4 was enriched with cadherin domains, SAND domain-like structures, MAD homology-1 domains, CTF transcription factor/nuclear factor domains, and NET domains (Figure 4D).

### Protein-complex and KEGG-pathway analysis of quantitatively altered phosphorylated proteins

**Figure 5:**
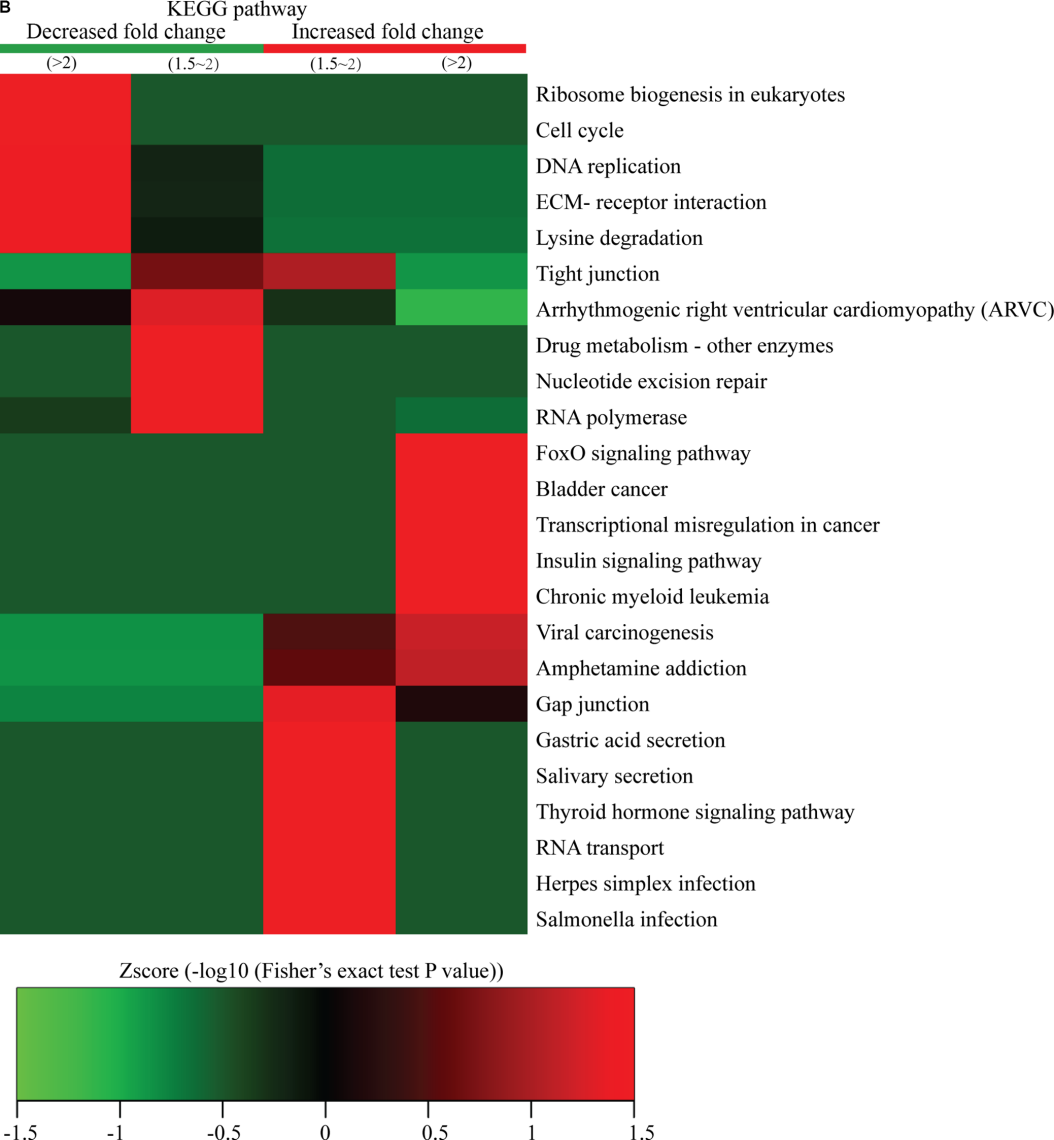
Protein-complex and KEGG-pathway analysis of quantitatively altered phosphorylated proteins The CORUM protein complex database for humans (http://mips.helmholtz-muenchen.de/genre/proj/corum/) was used for protein-complex analysis. The KEGG (http://www.genome.jp/kegg/) database was used to investigate enriched pathways using the DAVID Functional Annotation Tool against the background of *Homo sapiens*. (**A**) Protein complex analysis. (**B**) KEGG pathway analysis.

Protein-complex and KEGG-pathway analyses of quantitatively altered phosphorylated proteins were also performed. Protein-complex analysis of the quantitatively altered phosphorylated proteins revealed that Q1 was enriched with proteins involved in the DNA-synthesis and -repair core complex, while Q2 was enriched with proteins involved in the nucleolar-remodeling and MDC1 complexes. The proteins exhibiting increased phosphorylation events were mainly included in the large Drosha complex, MLL-HCF complex, the MecP1 complex in the Q3 quantile, while proteins involved in the ctBP complex, SAP complex, and ING2 complex were found to be enriched in Q4 quantile (Figure 5A).

We also performed KEGG-pathway analysis of the quantitatively altered proteins, with the results showing that pathways involved in ribosome biogenesis in eukaryotes, cell cycle, DNA replication, drug metabolism, nucleotide-excision repair, and RNA polymerase activity were highly represented in Q1- and Q2-quantile proteins exhibiting decreased lysine-phosphorylation events, while proteins exhibiting increased lysine-ubiquitination events were involved in cancer misregulation and digestive-system functions, such as gastric acid and salivary secretion (Figure 5B). These results suggested that proteins exhibiting increased phosphorylation levels following FK228 stimulation were highly associated with cell signaling activity and disease progression.

### Protein-protein interaction networks associate with differentially phosphorylated proteins

**Figure 6:**
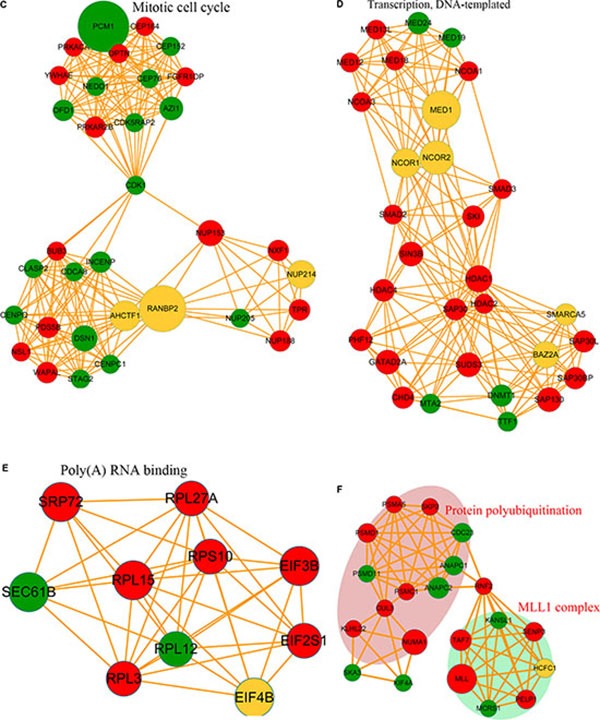
Phosphorylated protein-protein interaction network analysis The STRING database system (http://string-db.org/) was used to construct a protein-protein interaction network, and functional protein-interaction networks were visualized using Cytoscape (http://www.cytoscape.org/). (**A**) All identified phosphorylated protein-interaction network. (**B**) Spliceosome-related phosphorylated-protein-interaction network. (**C**) Mitotic cell cycle-related phosphorylated-protein-interaction network. (**D**) Transcription DNA-template-related phosphorylated-protein-interaction network. (**E**) Poly(A) RNA-binding-related phosphorylated-protein-interaction network. (**F**) Protein polyubiquitination and MLL1-complex-related phosphorylated-protein-interaction network.

Protein-interaction networks can serve as an alternative strategy for analysis of physical and functional interactions [35]. The protein-protein interaction networks associated with the differentially phosphorylated proteins were analyzed, with the results showing that proteins exhibiting increased lysine phosphorylation following FK228 treatment were highly enriched in the spliceosome. In contrast, proteins exhibiting decreases in lysine phosphorylation in response to FK228 treatment were involved in the mitotic cell cycle, transcription, DNA-template activity, Poly(A) RNA binding, protein polyubiquitination, and the MLL1 complex (Figure 6A). In the spliceosome-related phosphorylated-protein-interaction network, there were 16 sites exhibiting increases in modifications in response to FK228 treatment, two sites exhibiting decreased modification, and two sites exhibiting both increased and decreased modifications (Figure 6B). In contrast, dysregulated modification sites associated with the mitotic cell cycle-related phosphorylated-protein-interaction network included 16 sites exhibiting decreased modifications and 16 sites exhibiting increased modifications, with only two sites exhibiting both increased and decreased modifications (Figure 6C). The phosphorylated-protein-interaction networks associated with DNA-template-related transcription involved 20 sites exhibiting increased modifications and five sites exhibiting decreased modifications, five sites exhibiting both increased and decreased modifications (Figure 6D). The phosphorylated-protein-interaction networks associated with poly(A) RNA binding contained seven sites exhibiting increased modifications, two sites exhibiting decreased modifications, and one site exhibiting both increased and decreased modifications (Figure 6E). The phosphorylated-protein-interaction networks associated with polyubiquitination- and MLL1-complexes included 11 sites exhibiting increased modifications, eight sites exhibiting decreased modifications, and one site exhibiting both increased and decreased modifications (Figure 6F). All data are listed in Supplementary Table S3.

### Acetylome profile alterations following FK228 treatment

We investigated the acetylation levels of non-histone proteins in response to FK228 treatment using SILAC, Kac-antibody enrichment, and LC-MS/MS analysis. A total of 1289 Kac sites corresponding to 756 proteins were identified from HCT-8 and HCT-116 cells, and 1175 sites in 747 proteins were quantified (Supplementary Table S4). To the best of our knowledge, this is the first comprehensive profiling of Kac data following FK228 treatment in HCT-8 and HCT-116 cells.

### GO analysis and protein domains associated with quantitatively altered acetylated proteins

We performed enrichment-based clustering analyses to compare the functions of identified proteins exhibiting increased and decreased acetylation levels (Figure 5A–5F). For clustering analysis, all the quantified acetylation sites were also divided into four quantiles (Q1–Q4) according to L/H ratios as previously described.

**Figure 7:**
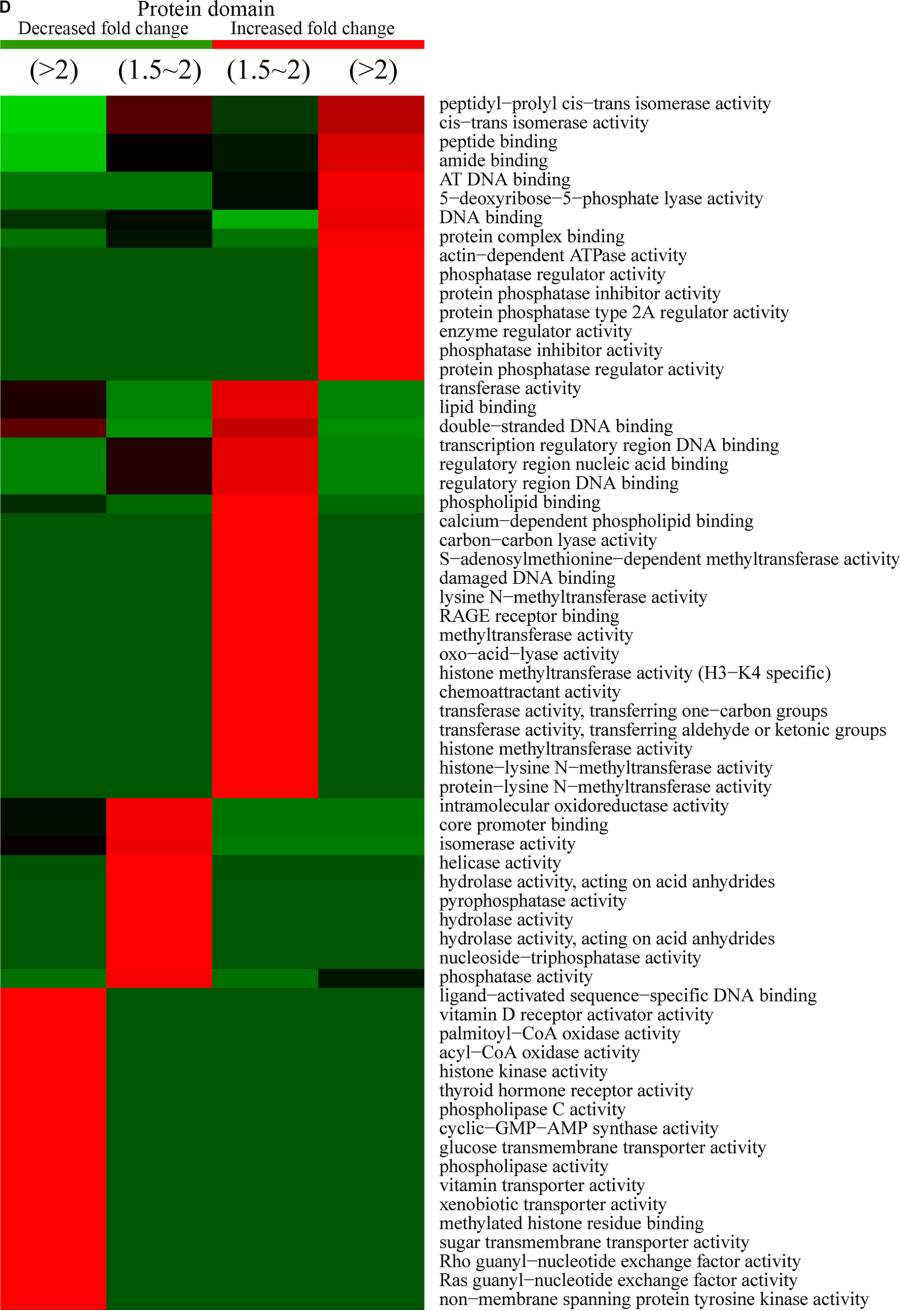
Functional enrichment-based clustering analysis for the quantified acetylated proteins According to the functional differences observed between proteins exhibiting increased or decreased modification sites, GO-term association and enrichment analysis using DAVID (https://david.ncifcrf.gov/) was performed. (**A**) Proteins involved in biological processes, (**B**) Cellular component processes, (**C**) molecular functions, and (**D**) protein domain processes.

The results of our analyses regarding the biological processes associated with acetylation are shown in Figure 7A. Proteins exhibiting decreased lysine acetylation were involved in the regulation of metabolic processes, including positive regulation of homeostatic processes, cholesterol regulation, ossification regulation, and response to organic substances, indicating that FK228 treatment can influence protein metabolism. Additionally, the functions of proteins exhibiting decreased Kac were also focused on cell signaling-transduction mechanisms, such as signaling, single-organism signaling, programmed cell death, extracellular matrix organization, and extracellular structure organization. Conversely, the proteins exhibiting increased Kac sites were involved in response to stress and regulation of organismal processes, including carbohydrate catabolic processes, antigen processing, presentation of peptide antigens, reproductive processes, and mitotic cell cycle regulation.

Cellular-component analysis showed that some proteins located on the membrane, such as Acyl-CoA *N*-acyltransferase, histidine kinase, and DNA topoisomerase, were highly enriched in Q1, while homeodomain-like domain-containing proteins, helicases, RanBP2-type zinc-finger domain-containing proteins, and DEAD/DEAH box-type DNA/RNA helicases were abundant in Q2 (Figure 7B). In contrast, increased Kac sites were found in ubiquitin-like domain-containing proteins, linker histone H1/H5, glutathione S-transferase, SET domain-containing proteins, post-SET domain-containing proteins, and MOZ/SAS-like proteins.

From molecular-function analysis (Figure 7C), we observed that proteins classified as members of the melanosome, pigment-granule proteins, and histone acetyltransferase complex-related proteins were enriched in the Q1 quantile. Catalytic step 2 spliceosome proteins, nucleolus proteins, and some complex-associated proteins were enriched in the Q2 quantile. In contrast, proteins exhibiting increased Kac sites in Q3 were classified as DNA-bending complexes, protein-DNA complexes, cortical cytoskeleton, spindle, chromatin, action cytoskeleton, cell projections, cytoskeleton, cell-cell adherens junction, and cell-junction proteins.

Following protein-domain analysis, we observed that the domains involved in ligand-activated sequence-specific DNA binding, oxidase activity, synthase activity, transporter activity, phospholipase C activity, and tyrosine kinase activity were enriched in Q1 proteins exhibiting decreased sites involving Kac modifications. The domains involved in oxidoreductase activity, core-promoter binding, isomerases, nucleoside-triphosphatases, helicases, hydrolases, and phosphatase activity were enriched in Q2 proteins. The proteins exhibiting increased Kac-modification sites in Q3 contained domains involving transferase activity, DNA binding, lyase activity, methyltransferase activity, and transferase activity. Domains involved in isomerase activity, cis-trans isomerase activity, peptide binding, amide binding, DNA binding, protein-complex binding, actin-dependent ATPase activity, phosphatase-regulator activity, protein phosphatase-inhibitor activity, and protein phosphatase-regulator activity were abundant in Q4 proteins (Figure 7D).

### Protein-complex and KEGG-pathway analysis of quantitatively altered acetylated proteins

**Figure 8:**
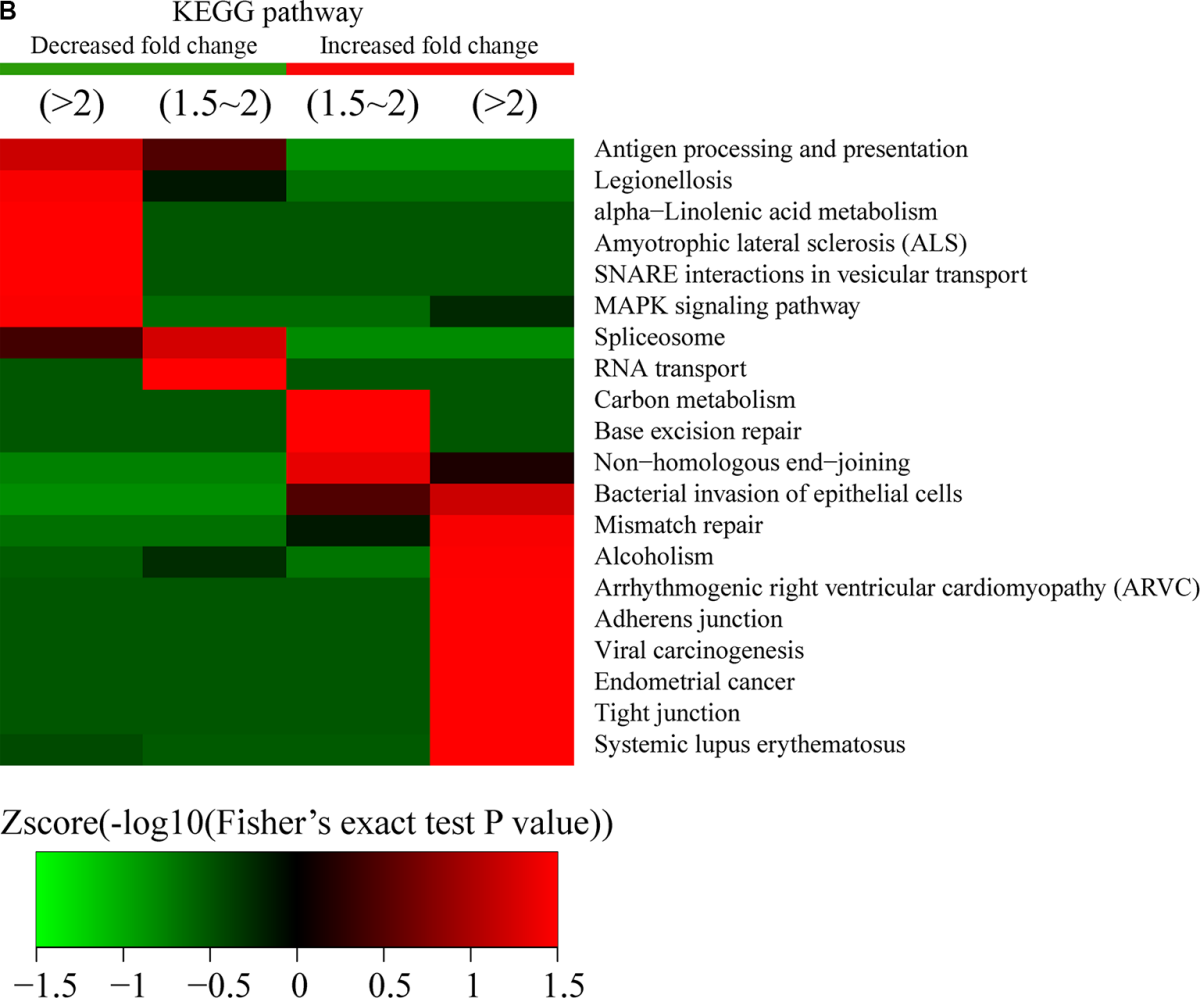
Protein-complex and KEGG-pathway analysis of quantitatively altered acetylated proteins Methods used for analysis were similar to those described for the proteins exhibiting altered phosphorylation levels. (**A**) Protein complex analysis. (**B**) KEGG pathway analysis.

Protein-complex analysis of the quantitatively altered acetylated proteins (Figure 8A) showed that proteins involved in the HBO1 complex and emerin complex were enriched in proteins exhibiting decreased Kac-modification sites. The 53 BP1-containing complexes, RNA poly II-containing coactivator complexes, Tat-SF complexes, G2/M-phase of the cell cycle, histone H3.1 complexes, and DNA-PK-Ku involved proteins exhibiting increased Kac-modification sites.

KEGG-pathway analysis of the quantitatively altered proteins showed that pathways involving antigen processing and presentation, legionellosis, alpha-linolenic acid metabolism, amyotrophic lateral sclerosis, SNARE correlates in vesicular transport, the MAPK-signaling pathway, the spliceosome, and RNA transport were enriched in Q1 and Q2 proteins, while proteins exhibiting increased Kac-modification sites were involved in carbon metabolism, base-excision repair, mismatch repair, alcoholism, arrhythmogenic right-ventricular cardiomyopathy, adherens junction activity, viral carcinogenesis, endometrial cancer, tight-junction activity, and systemic lupus erythematosus (Figure 8B). These results showed that acetylation was highly associated with cell signaling and diseases.

### Protein-protein interaction network analysis of the differentially acetylated proteins

**Figure 9:**
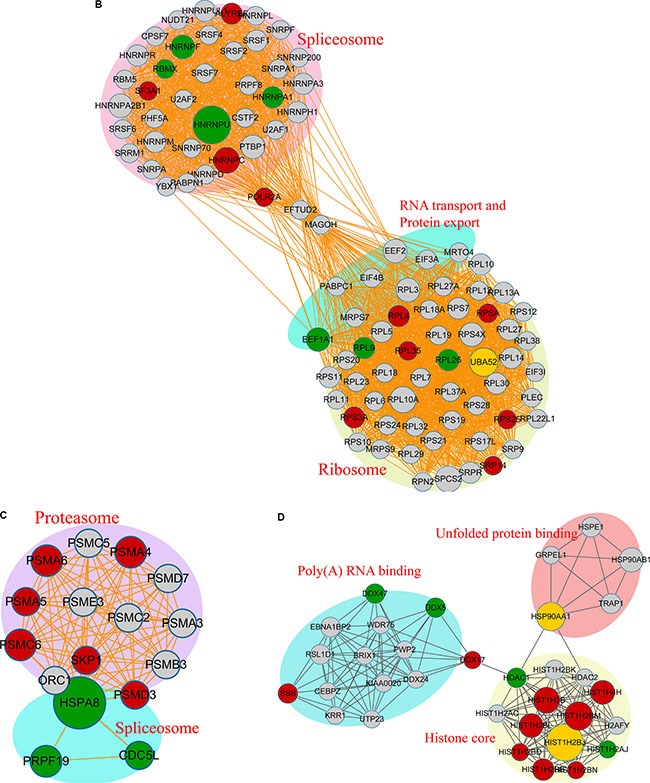
Acetylated protein-protein interaction analysis The STRING database system (http://string-db.org/) was used to construct a protein-protein interaction network, and functional protein-interaction networks were visualized using Cytoscape (http://www.cytoscape.org/). (**A**) All identified acetylated protein-interaction networks. (**B**) The acetylated-protein-interaction network associated with the spliceosome, ribosome, RNA transport, and protein export. (**C**) The acetylated-protein-interaction network associated with the proteasome and spliceosome. (**D**) The acetylated-protein-interaction network associated with unfolded-protein binding, Poly(A) RNA binding, and the histone core.

We next constructed a protein-protein interaction network for the proteins containing Kac sites. Our results revealed that proteins with Kac sites were involved in the spliceosome, ribosome, and RNA transport, while dysregulation of protein export, the proteasome, unfolded-protein binding, poly(A) RNA binding, and the histone core was observed in response to FK228 treatment was observed (Figure 9A). In the acetylated-protein-interaction network associated with the spliceosome, there were 10 modification sites exhibiting increased acetylation, seven modification sites exhibiting decreased acetylation, and one site exhibiting both increased and decreased acetylation (Figure 9B). The proteasome- and spliceosome-related networks associated with proteins exhibiting dysregulated acetylation included six modification sites exhibiting increased acetylation and three modification sites exhibiting decreased acetylation, respectively (Figure 9C). The acetylated-protein networks related to unfolded-protein binding, poly (A) RNA binding, and histone core included nine modification sites exhibiting increased acetylation and four modification sites exhibiting decreased modification, with two sites exhibiting both increased and decreased modifications (Figure 9D). These data are listed in Supplementary Table S5.

### Crosstalk between quantitated phosphorylation and the acetylome

**Figure 10:**
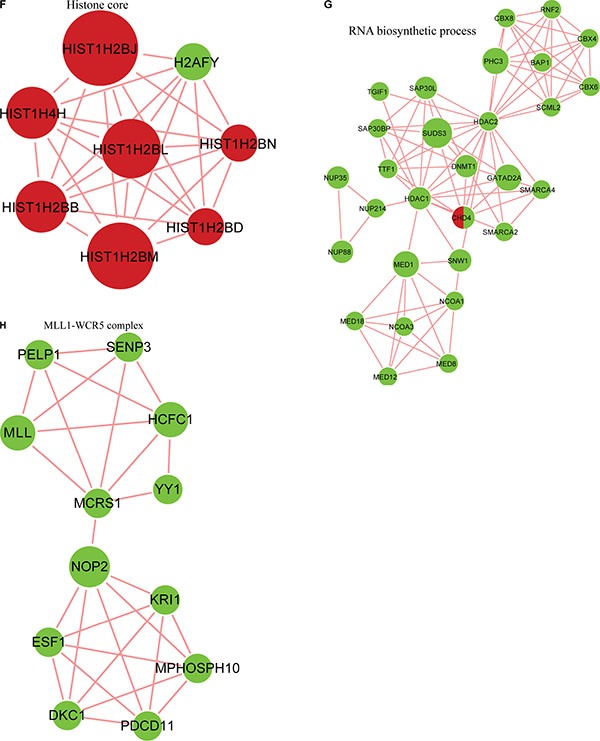
Analysis of the crosstalk between phosphorylation levels and the acetylome (**A**) Overlap between phosphorylated proteins and acetylated proteins. Protein-protein interaction network between phosphorylated proteins and acetylated proteins obtained from Cytoscape (**B**), in the spliceosome (**C**), the nucleoplasm and mitotic prometaphase (**D**), in G2/M transition of the mitotic cell cycle and nuclear-transcribed mRNA catabolic processes (**E**), in the histone core(F),in RNA-biosynthetic processes (**G**) and clustered in the MLL1-WDR5 complex (**H**).

To study the crosstalk between acetylation and phosphorylation, we compared the data from the acetylome with global phosphorylation data obtained from FK228-treated HCT-8 and HCT-116 cells and identified 274 proteins that underwent both acetylation and phosphorylation (Figure 10A). These proteins exhibited consistent changes in modification, suggesting positive correlations between acetylation and phosphorylation in these proteins.

**Figure 11:**
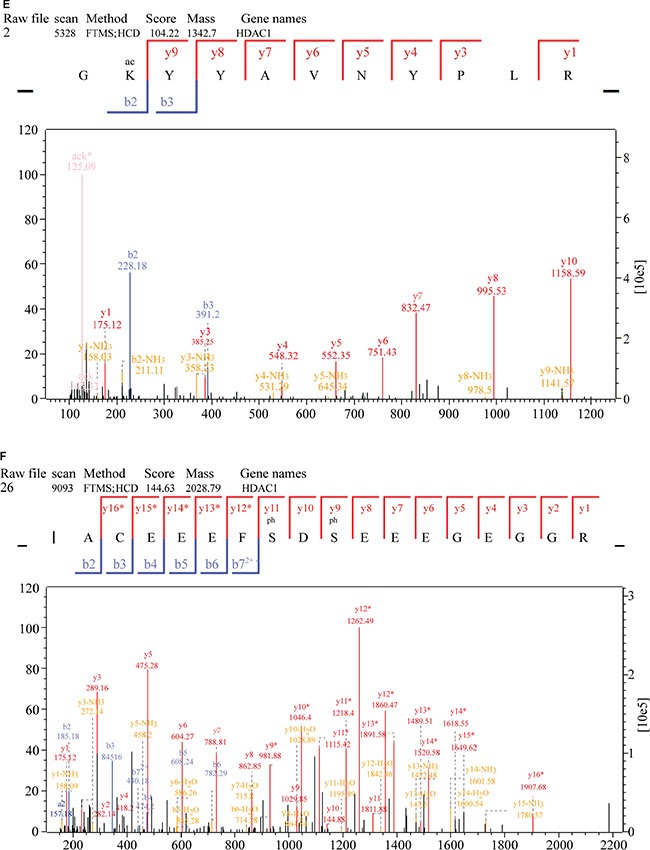
MS/MS spectra of P08238, Q8NEZ4, and Q13547 acetylation and phosphorylation (**A**) P08238 acetylation, (**B**) P08238 phosphorylation, (**C**) Q8NEZ4 acetylation, (**D**) Q8NEZ4 phosphorylation, (**E**) Q13547 acetylation, (**F**) Q13547 phosphorylation. These proteins were underwent both acetylation and phosphorylation.

To further investigate potential crosstalk between the acetylome and phosphorylation states, a protein-protein interaction network based on Kac and lysine-phosphorylated proteins was constructed (Figure 10B). The global overview of the protein-protein interaction network between Kac and lysine-phosphorylated proteins was obtained (Figure 10B), followed by clustering these proteins according to multiple biological processes (Figure 10C). We observed that Kac and lysine-phosphorylated proteins both participated in the spliceosome, nucleoplasm activity, mitotic prometaphase, G2/M transition of the mitotic cell cycle, nuclear-transcribed mRNA catabolic processes, histone core interactions, RNA-biosynthetic processes, and MLL1-WDR5 complexes (Figure 10D–10H; Supplementary Table S6). Some important proteins involved in these networks were both acetylated and phosphorylated, including HNRNP A1, HNRNP U, and the CHD4 complex. These proteins might be important in mediating the effects of FK228 treatment of HCT-8 and HCT-116 cells, and, therefore, their biological functions in cancer should be further investigated. The representative spectra of P08238, Q8NEZ4, Q13547, Q01105, O14617, Q9Y3Z3, Q6ZU65, P18583, Q8WUA2, P20810 proteins that underwent both acetylation and phosphorylation are presented in Figure 11 and Supplementary Figure S1.

## DISCUSSION

Protein function is regulated by diverse PTMs, which represent a key means of regulating cellular processes and diversifying the proteome, resulting in regulation of protein activity, subcellular localization, and maintenance of homeostasis. Modification of specific protein residues can confer different regulatory outcomes individually or in a combinatorial manner [32]. Phosphorylation and acetylation are the two most prevalent PTMs observed in the eukaryotic proteome. Phosphorylation is the primary mechanism involved in cellular signaling, while acetylation plays a prominent role in protein expression, and can inhibit protein degradation. Phosphorylation and acetylation of lysine residues represent prominent and ubiquitous regulatory mechanisms that are conserved throughout the human proteome [33–38]. For example, van Noort et al. [39] reported that phosphorylation and lysine acetylation could be simultaneously measured in the small bacterium *Mycoplasma pneumoniae*. Cheung et al. [40] found that histone H3 phosphorylation and acetylation are synergistic in response to epidermal growth factor stimulation.

Here, we determined both the quantitative proteome and the acetylome in HCT-8 and HCT-116 cells following FK228 treatment. We found that FK228 treatment altered protein acetylation levels, resulting in both increases and decreases in acetylation events. The quantitative acetylome from HCT-8 and HCT-116 cells following FK228 treatment was also obtained, with > 1000 Kac sites quantified. From these sites, modifications to 115 lysine-acetylation sites in 85 proteins were quantified as being increased and modifications to 38 sites in 32 proteins were decreased in response to FK228 treatment. Interestingly, 47 histone lysine-acetylation sites were identified in core histone proteins. These significantly altered proteins are involved in multiple biological functions, as well as a myriad of metabolic and enzyme-regulated pathways. For these interesting events, further study should be performed to elucidate the associated mechanisms.

Quantitative phosphoproteomics analysis was also performed in HCT-8 and HCT-116 cells following FK228 treatment. We identified 4982 phosphorylation sites in 2062 protein groups in total, among which, 4166 sites in 1937 proteins were quantified. When setting a quantification ratio of > 2.0 as the threshold for sites exhibiting increased modifications and < 0.5 as the threshold for sites exhibiting decreased modifications, 901 phosphorylation sites in 603 proteins were quantified as exhibiting increased modifications and 375 sites in 292 proteins were quantified as exhibiting decreased modifications. Finally, the crosstalk between Kac and phosphorylation was also analyzed. According to our results, Kac and lysine-phosphorylated proteins both participated in the spliceosome, nucleoplasm activity, mitotic prometaphase, G2/M transition of the mitotic cell cycle, histone core interactions, RNA-biosynthetic processes, and MLL1-WDR5 complexes. Some important proteins involved in these networks were both acetylated and phosphorylated, including coexistence of phosphorylation and Kac in hnRNPA1, hnRNPU, MKI67, sAFB, lMNB1, SET, MAP4, SKP1, PARVA, AP3D1, SAMHD1, RFC1, UBN2, TOP2A, SON, CTNNA1, PPIL4, CAST, NOLC1, and CHD4. These proteins might be important in mediating the effects of FK228 treatment of cancer cells, and, therefore, their biological functions in cancer should be further investigated.

Wu et al. [27, 28] investigated the quantitative proteome, acetylome, and ubiquitylome, as well as crosstalk between the three datasets in A549 cells following SAHA treatment. Their results identified 55 common sites exhibiting both acetylation and ubiquitination. Sakaguchi et al. [41] reported that p53 is acetylated *in vitro* at separate sites by two different histone acetyltransferases, suggesting that DNA damage enhances p53 activity as a transcription factor in part through carboxy-terminal acetylation that is directed by N-terminal phosphorylation. Wang et al. [42] showed that an ATM- and Rad3-related signaling pathway and a phosphorylation-acetylation cascade are involved in activation of p53/p21Waf1/Cip1 in response to 5-aza-2′-deoxycytidine treatment. Chen et al. [43] revealed that the acetylation of RelA at Lys310 is regulated by prior phosphorylation of Ser276 and Ser536. Such phosphorylated and acetylated forms of RelA display enhanced transcriptional activity [43].

According to the results presented here, FK228 treatment directly altered lysine acetylation and phosphorylation levels in HCT-8 and HCT-116 cells. Moreover, the alteration of acetylation and phosphorylation levels also ultimately regulate each other due to the existing crosstalk between acetylation and phosphorylation events.

Additionally, the changing levels of lysine acetylation could induce alterations in the global proteome. Moreover, transcription factors could also undergo phosphorylation and induce alterations to the global proteome via the transcriptome. However, these constitute hypotheses and require confirmation through additional experimentation.

In conclusion, using SILAC labeling, antibody-based affinity enrichment, and high-resolution LC-MS/MS, we obtained results suggested that FK228 treatment broadly altered the proteome, phosphorylation levels, and the acetylome in HCT-8 and HCT-116 cells. Additionally, potential crosstalk between phosphorylation states and the acetylome associated with FK228 treatment were observed through bioinformatics analyses, which expanded our current understanding of FK228-related cancer therapy. We noted potential interactions between the acetylome, phosphorylation levels, and the proteome based on positive regulation between the acetylome and phosphorylation events. However, further experiments are necessary to enhance validation and interpretation of the predicted mechanisms.

## MATERIALS AND METHODS

### Cell culture

HCT-8 and HCT-116 colon cancer cells were purchased from the Cell Bank at the Chinese Academy of Sciences (Shanghai, China). The cells were cultured in glucose (4.5 g/L) and Dulbecco's Modified Eagle Medium (DMEM) supplemented with glutamine and sodium pyruvate and containing 10% fetal bovine serum (FBS) and 1% penicillin-streptomycin at 37°C with 95% air and 5% CO_2_. The cells were subcultured every 2 to 3 days after digestion with 0.02% ethylenediaminetetraacetic acid (EDTA) and 0.1% trypsin.

### Cell cytotoxicity assay

Previous reports indicated that 50% of HCT-8 cells were viable (IC_50_) following administration of FK228 at concentrations of 29.46 nM [30]. We performed cytotoxicity assays on HCT-116 cells using varying concentrations of FK228 according to previously described methods [30]. When HCT-116 cells reached 75% confluence, a cell proliferation and cytotoxicity assay was performed using Cell Counting Kit-8 (CCK-8; Dojindo Laboratories, Kumamoto, Japan) according to manufacturer instructions. Briefly, a 96-well plate was pre-incubated for 24 h, followed by addition of different concentrations of FK228 (Sigma-Aldrich, St. Louis, MO, USA) to each well. CCK-8 solution (10 μL) was added and incubated for an additional 1 to 4 h. Cell viability was determined by measuring the absorbance at 450 nm. Cell cytotoxicity was calculated using the HTC-116 cell-viability measurements following treatment with FK228 The FK228 concentration resulting in 50% cell viability (IC_50_) was used as the fixed working concentration for later experiments.

### SILAC labeling and FK228 treatment

Cells were grown to 80% confluence and labeled with either “heavy isotopic lysine” (13C-Lysine) or “light isotopic lysine” (12C-Lysine) using a SILAC protein quantitation kit (Pierce; Thermo Scientific, Rockford, IL, USA) according to manufacturer instructions. Briefly, cells were grown in DMEM supplemented with 10% FBS and either the “heavy” form of [U-13C6]-l-lysine or “light” [U-12C6]-l-lysine for more than six generations in order to achieve > 97% labeling efficiency before being harvested, following by expanding in SILAC media to achieve the ~5 × 10^8^ cell number in 150-cm^2^ flasks.

The “light”-labeled cells were treated with 29.46 nM FK228, and the “heavy”-labeled cells were treated with same concentration of dimethyl sulfoxide. After treatment, the cells were maintained in SILAC media for another 48 h before being harvested and washed twice with ice-cold phosphate-buffered saline (PBS) containg 2 μM trichostatin A and 30 mM nicotinamide. After freezing in liquid nitrogen, cell pellets were stored at −80°C until further use.

### Protein extraction

The harvested “heavy”- and “light”-labeled cells were lysed with 2× NETN buffer [200 mM NaCl, 100 mM Tris-Cl, 2 mM EDTA, and 1.0% NP-40 (pH 7.2)] supplemented with 0.5% Triton X-100 on ice for 30 min. The supernatant was saved following centrifugation at 20,000 g for 10 min at 4°C. After concentration measurement, equal amounts of crude proteins from the “heavy”- or “light”-labeled supernatant were mixed and precipitated by adding trifluoroacetic acid (TFA) to a 15% (v/v) final concentration (soluble fraction). After washing twice with −20°C acetone, the protein pellets were dissolved in 100 mM NH_4_HCO_3_ (pH 8.0) for trypsin digestion. The remaining cell pellets were dissolved in 8 M urea to extract the chromatin-binding proteins. After measurement of protein concentration, equal amounts of chromatin-binding proteins in the urea solution were mixed, and the proteins were precipitated by adding TFA to a 15% (v/v) final concentration (nuclear-pellet fraction). After washing twice with −20°C acetone, the protein pellets were dissolved in 100 mM NH_4_HCO_3_ for trypsin digestion.

### Trypsin digestion

Protein solutions containing 1:50 (w/w) ratio of trypsin (Promega, Madison, WI, USA) to protein was prepared and digested at 37°C for 16 h. Dithiothreitol was added to a final concentration of 5 mM, incubated at 50°C for 30 min. In order to alkylate proteins, indole-3-acetic acid was added to a final concentration of 15 mM, and incubated in the dark for 30 min. The alkylation reaction was quenched by 30 mM cysteine for another 30 min. Trypsin was then added at a trypsin-to-protein ratio of 1:100 (w/w) for digestion at 37°C for 4 h to complete the digestion cycle.

### High-performance liquid chromatography (HPLC) fractionation

The sample was then fractionated by high pH, reversed-phase HPLC using an Agilent 300 Extend C18 column (5-μm particles, 4.6-mm internal diameter, 250-mm length; Agilent Technologies, Santa Clara, CA, USA). Peptides were initially separated into 80 fractions using a gradient of 2% to 60% acetonitrile in 10 mM ammonium bicarbonate (pH 10) over 80 min, followed by combining the peptides into six fractions and drying by vacuum centrifugation.

### Affinity enrichment

Before affinity enrichment, anti-lysine-acetylation (anti-Kac) and anti-lysine-phosphorylation antibody beads (PTM Biolabs, Inc., Hangzhou, China) were washed twice with ice-cold PBS. To enrich Kac and lysine-phosphorylated peptides, tryptic peptides bound to Kac and lysine-phosphorylation antibody beads dissolved in NETN buffer were incubated with pre-washed anti-Kac and anti-lysine-phosphorylated antibody beads at a ratio of 15 mL beads/mg protein at 4°C overnight with gentle shaking. The beads were washed four times with NETN buffer and twice with ddH_2_O. The bound peptides were eluted from the beads with 0.1% TFA, and the eluted fractions were combined, vacuum-dried, and analyzed by LC-MS/MS.

### LC-MS/MS analysis

Peptides were dissolved in 0.1% formic acid (FA) and directly loaded onto a reversed-phase pre-column (Acclaim PepMap 100; Thermo Fisher Scientific, Danvers, MA, USA). Peptide separation was performed using a reversed-phase analytical column (Acclaim PepMap RSLC; Thermo Fisher Scientific). The gradient comprised of an increase from 6% to 22% solvent B (0.1% FA in 98% acetonitrile) for 24 min, 22% to 35% for 10 min, a climb to 80% in 5 min, then holding at 80% for the last 3 min, all at a constant flow rate of 300 mL/min on an EASY-nLC 1000 UPLC system (Thermo Fisher Scientific). The resulting peptides were analyzed using a Q ExactiveTM hybrid quadrupole-Orbitrap mass spectrometer (Thermo Fisher Scientific).

The fractionated peptides were subjected to a nanospray ionization source, followed by MS/MS using the Q ExactiveTM hybrid quadrupole-Orbitrap mass spectrometer (Thermo Fisher Scientific) connected online to the UPLC system. Intact peptides were detected at a resolution of 70,000. Peptides were selected for MS/MS using a normalized collision energy setting of 28, and ion fragments were detected at a resolution of 17,500. A data-dependent procedure that alternated between one MS scan, followed by 20 MS/MS scans, was applied for the top 20 precursor ions above a threshold ion count of 2E4 in the MS survey scan, with a 15.0-s dynamic exclusion. The electrospray voltage applied was 2.0 kV. Automatic gain control was used to prevent overfilling of the ion trap, and 5E4 ions were accumulated for generation of the MS/MS spectra. For MS scans, the m/z scan range was 350 to 1800.

### Database search

The resulting MS/MS data were processed using MaxQuant with an integrated Andromeda search engine (v.1.4.1.2; http://www.biochem.mpg.de/5111795/maxquant). Tandem mass spectra were searched against the SwissProt database (20,274 sequences) concatenated with a reverse-decoy database. Trypsin/P was specified as the cleavage enzyme, allowing up to three missing cleavages, five modifications per peptide, and five charges. Mass error was set to 10 ppm for precursor ions and 0.02 Da for fragment ions. Carbamidomethylation on Cys was specified as a fixed modification, and oxidation on Met, acetylation on Lys, and acetylation on the protein N-terminus were specified as variable modifications. False discovery rate thresholds for protein, peptide, and modification sites were specified at 1%, and the minimum peptide length was set at 7 residues. All other parameters in MaxQuant were set to their default values. The site-localization probability was set at > 0.75.

### Bioinformatics analysis

We performed Gene Ontology (GO) term association and enrichment analysis using the Database for Annotation and Visualization and Integrated Discovery (DAVID; https://david.ncifcrf.gov/). The Kyoto Encyclopedia of Genes and Genomes (KEGG; http://www.genome.jp/kegg/) database was used to investigate enriched pathways using the DAVID Functional Annotation Tool against the background of *Homo sapiens*. Additionally, the InterPro database (http://www.ebi.ac.uk/interpro/) was searched using the DAVID Functional Annotation Tool against the background of *Homo sapiens*. The manually curated CORUM protein complex database for humans (http://mips.helmholtz-muenchen.de/genre/proj/corum/) was used for protein-complex analysis. The STRING database system (http://string-db.org/) was used to construct a protein-protein interaction network, and functional protein-protein interaction networks were visualized using Cytoscape (http://www.cytoscape.org/). When performing the bioinformatics analysis, a corrected *p* = 0.05 was considered significant.

## SUPPLEMENTARY MATERIALS FIGURES AND TABLES














