# Supplementary material for: Crosstalk among the proteome, lysine phosphorylation, and acetylation in romidepsin-treated colon cancer cells
Source: Oncotarget. 2016 Jul 26;7(33):53471–501. doi: 10.18632/oncotarget.10840 (PMC5288200; doi:10.18632/oncotarget.10840)
Supplement: Supplementary file 1 [file oncotarget-07-53471-s001.pdf]

Crosstalk among the proteome, lysine phosphorylation, and acetylation in romidepsin-treated colon cancer cells

Supplementary Materials

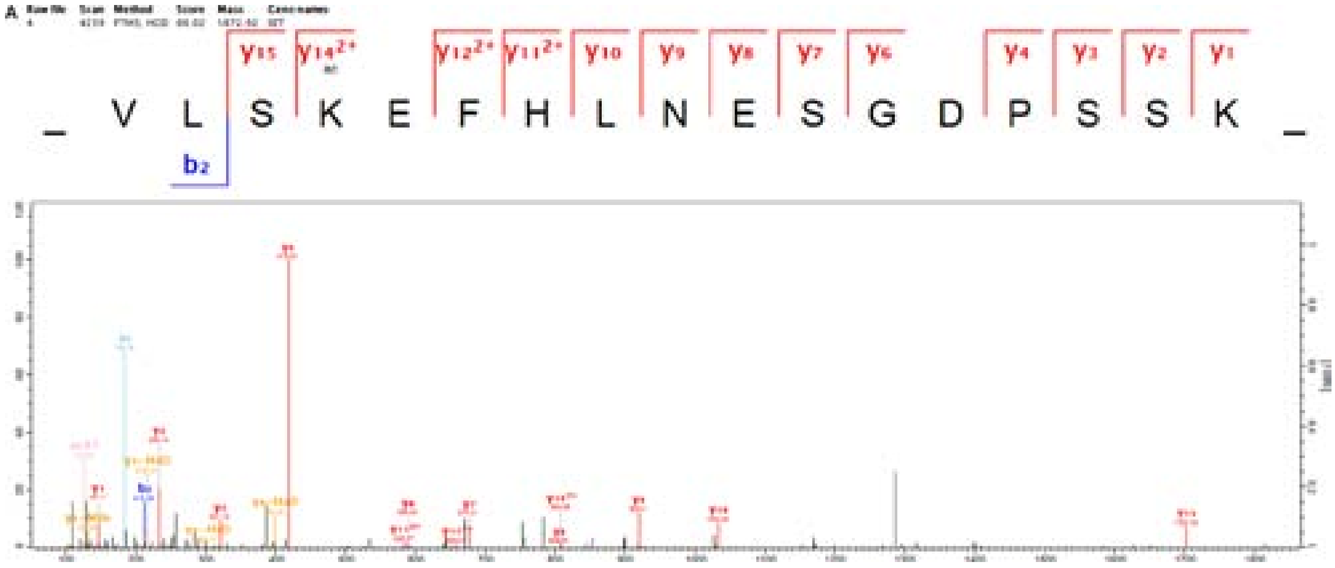

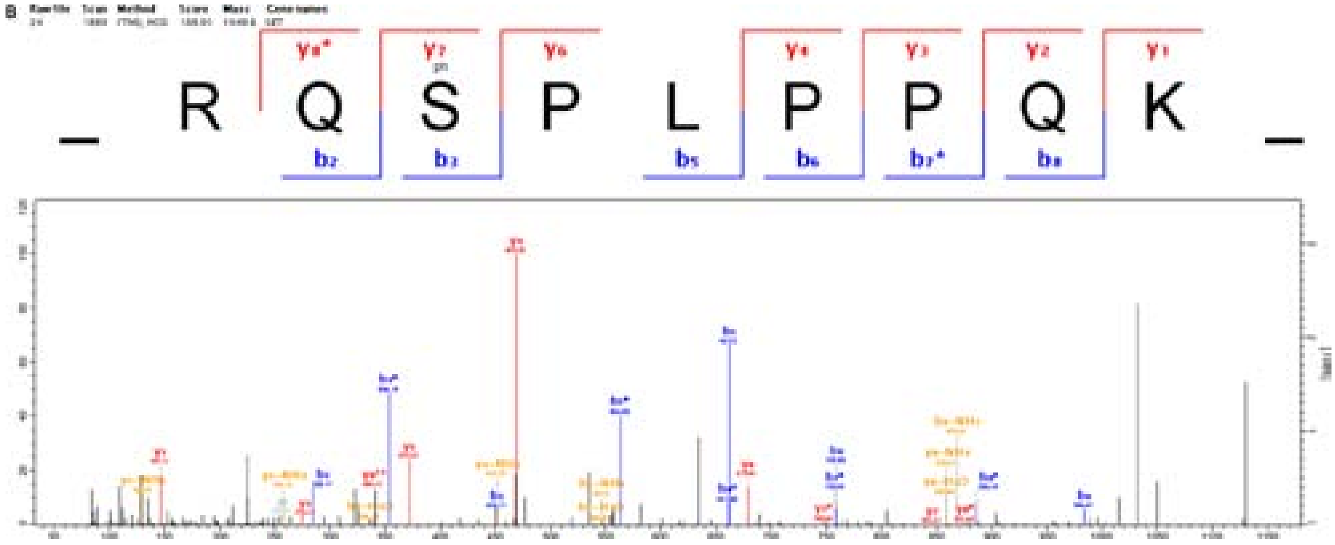

C

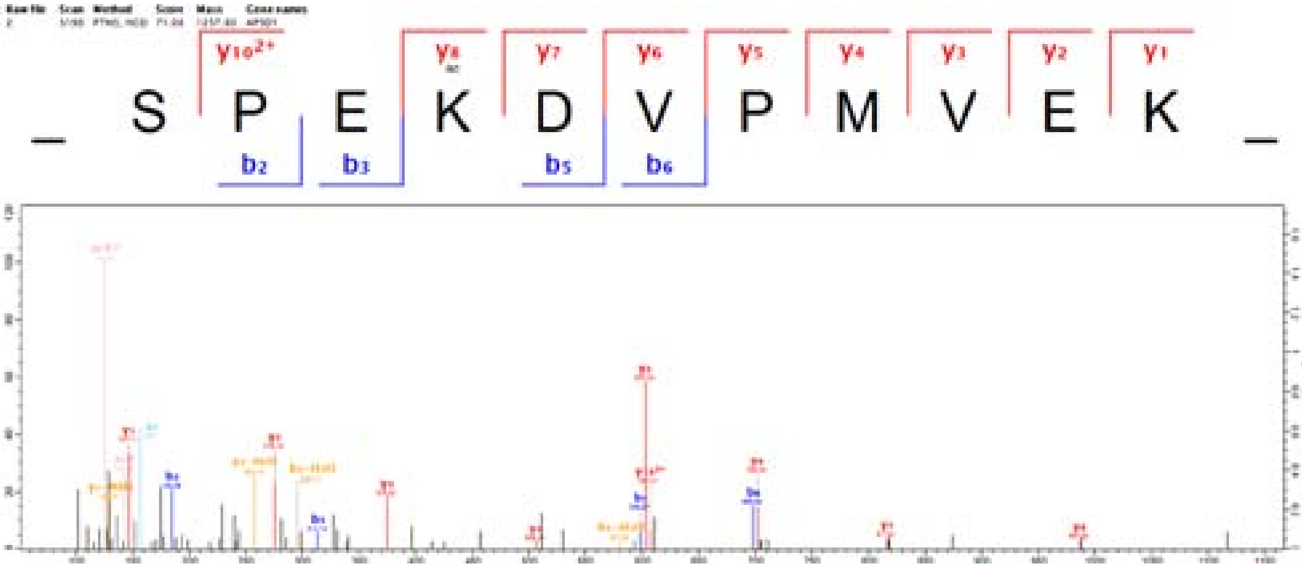

Raw file Scan Method Score Mass Gene names
FTMS; HCD
_ S P E K D V P M V E K _
y10 2+ y8 y7 y6 y5 y4 y3 y2 y1
b2 b3 b5 b6

D

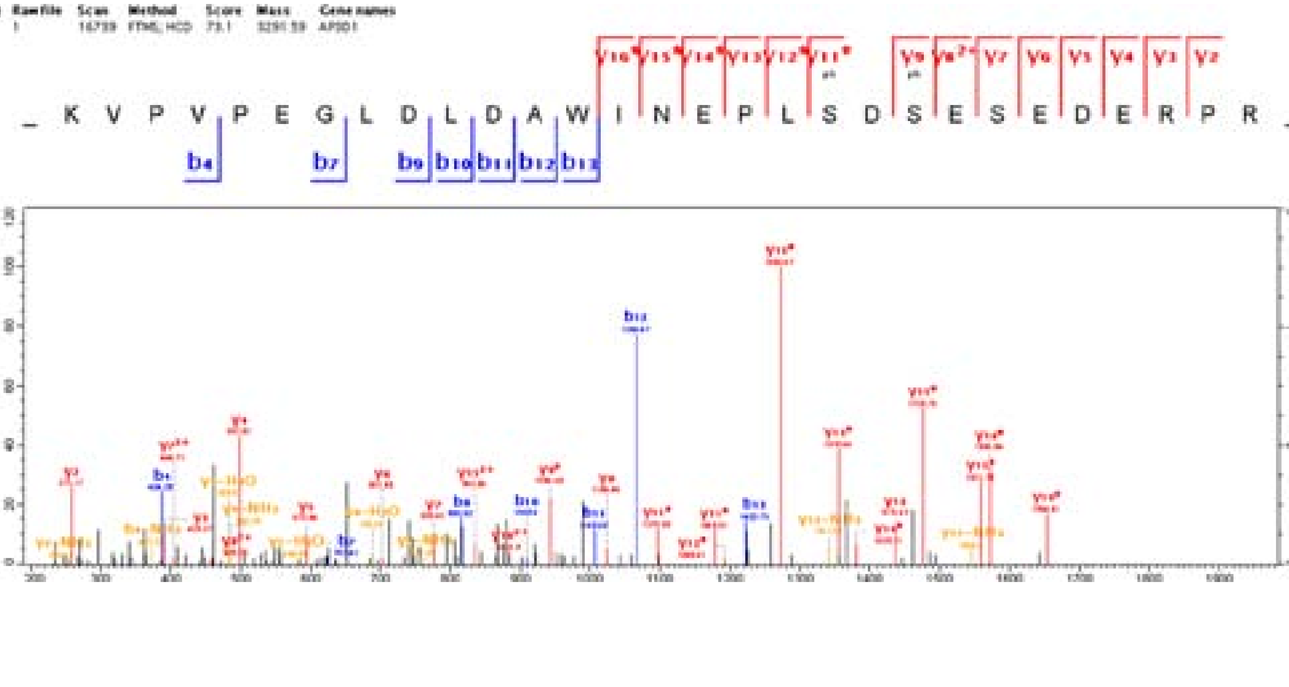

Raw file Scan Method Score Mass Gene names
1 16739 FTMS; HCD 79.1 3291.59 AP3D1
_ K V P V P E G L D L D A W I N E P L S D S E S E D E R P R _
y16 y15 y14 y13 y12 y11 y9 y8 2+ y7 y6 y5 y4 y3 y2
b4 b7 b9 b10 b11 b12 b13

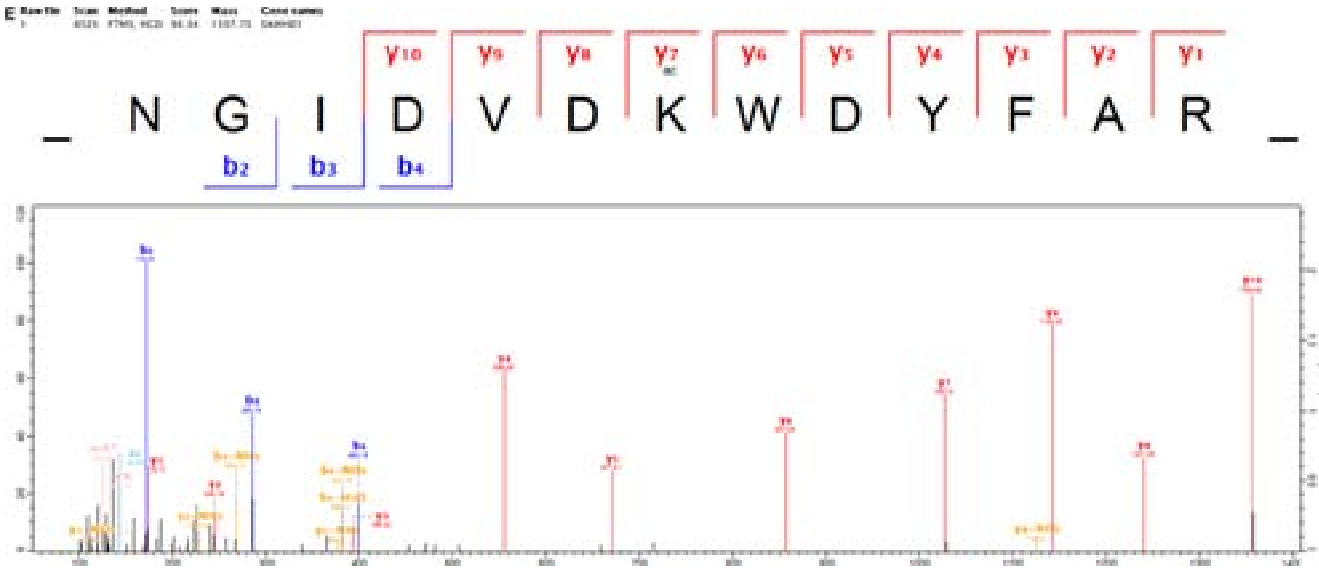
E
Raw file Scan Method Score Mass Gene names
_ N G I D V D K W D Y F A R _
y10 y9 y8 y7 y6 y5 y4 y3 y2 y1
b2 b3 b4

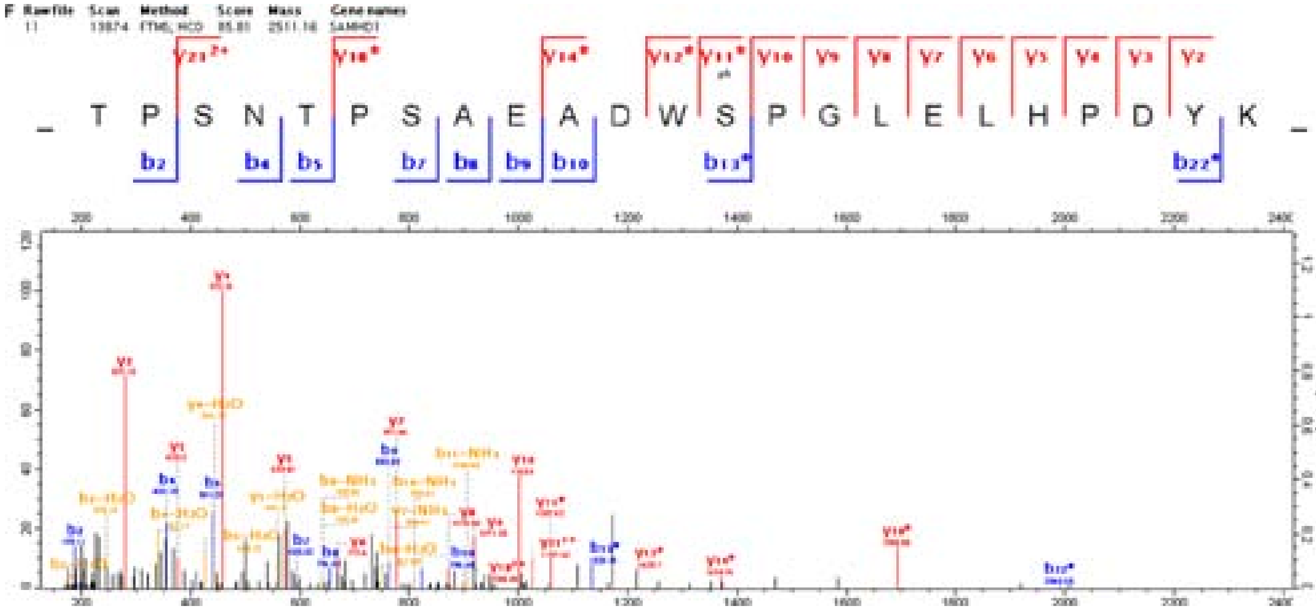
F
Raw file Scan Method Score Mass Gene names
11 13874 FTMS, HCD 85.81 2511.16 SAMHD1
_ T P S N T P S A E A D W S P G L E L H P D Y K _
y21 2+ y18* y14* y12* y11* y10 y9 y8 y7 y6 y5 y4 y3 y2
b2 b4 b5 b7 b8 b9 b10 b13* b22*

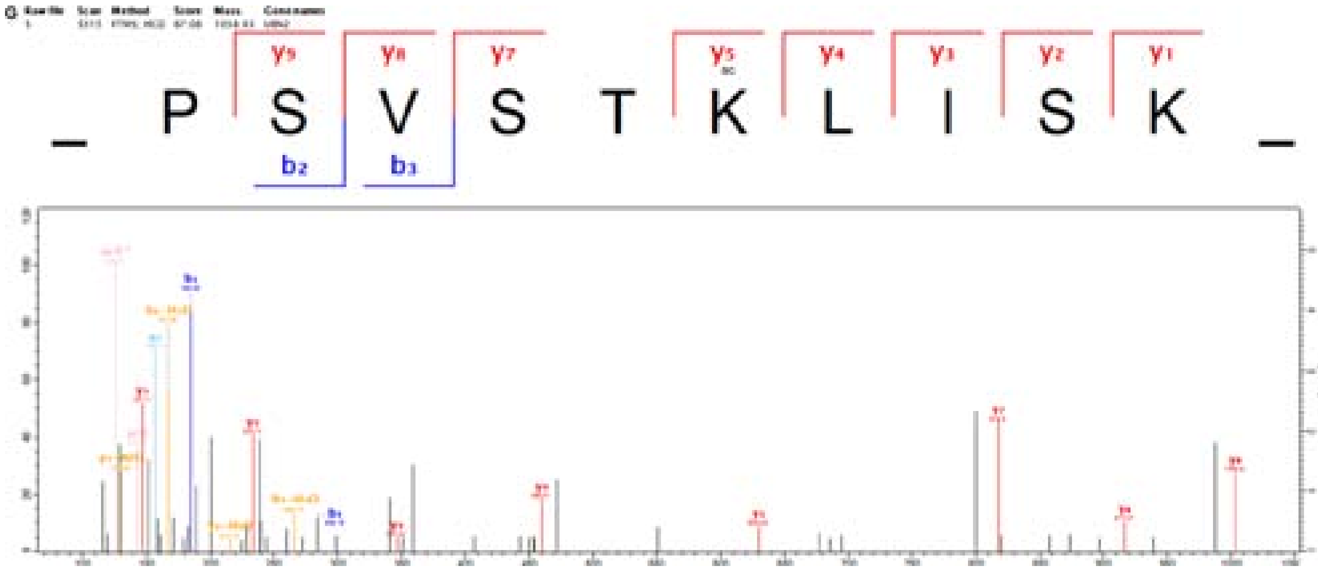
G
Raw file
Scan
Method
Score
Mass
Gene names
y9 y8 y7 y5 y4 y3 y2 y1
_ P S V S T K L I S K _
b2 b3

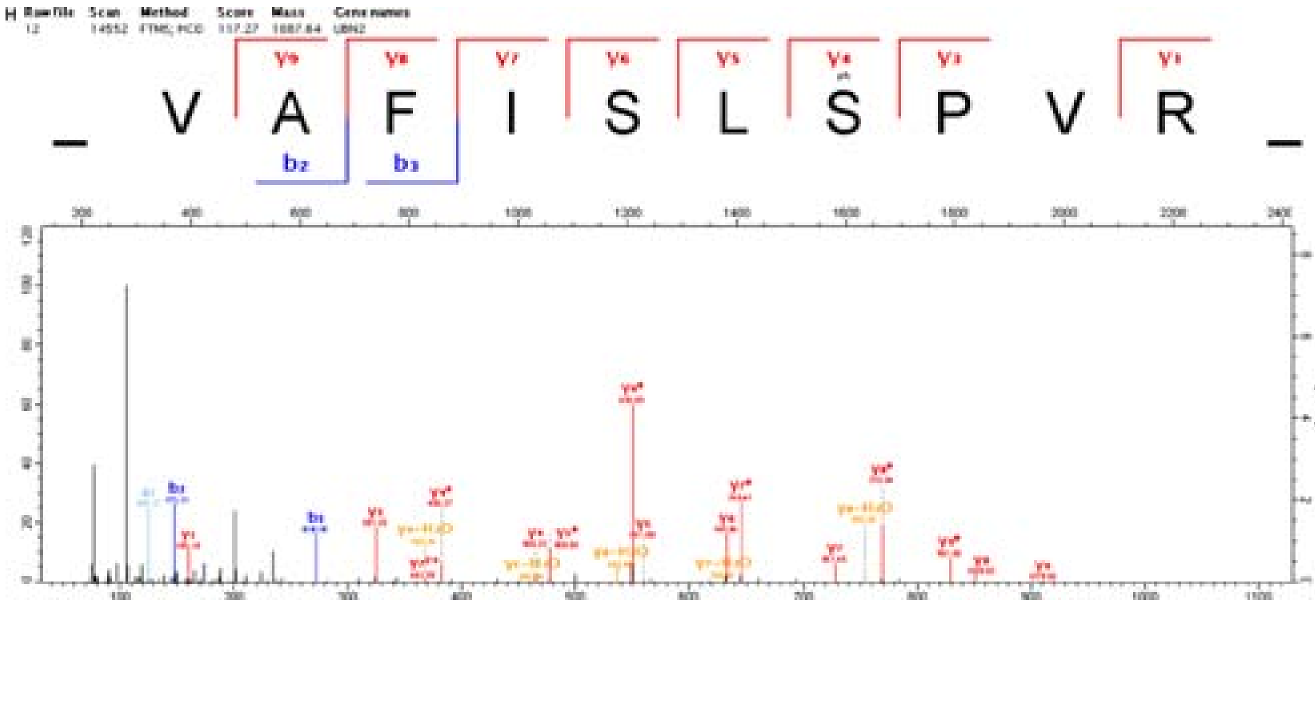
H
Raw file Scan Method Score Mass Gene names
12 14552 FTMS; HCD 117.27 1687.64 UBN2
y9 y8 y7 y6 y5 y4 y3 y1
_ V A F I S L S P V R _
b2 b3

I

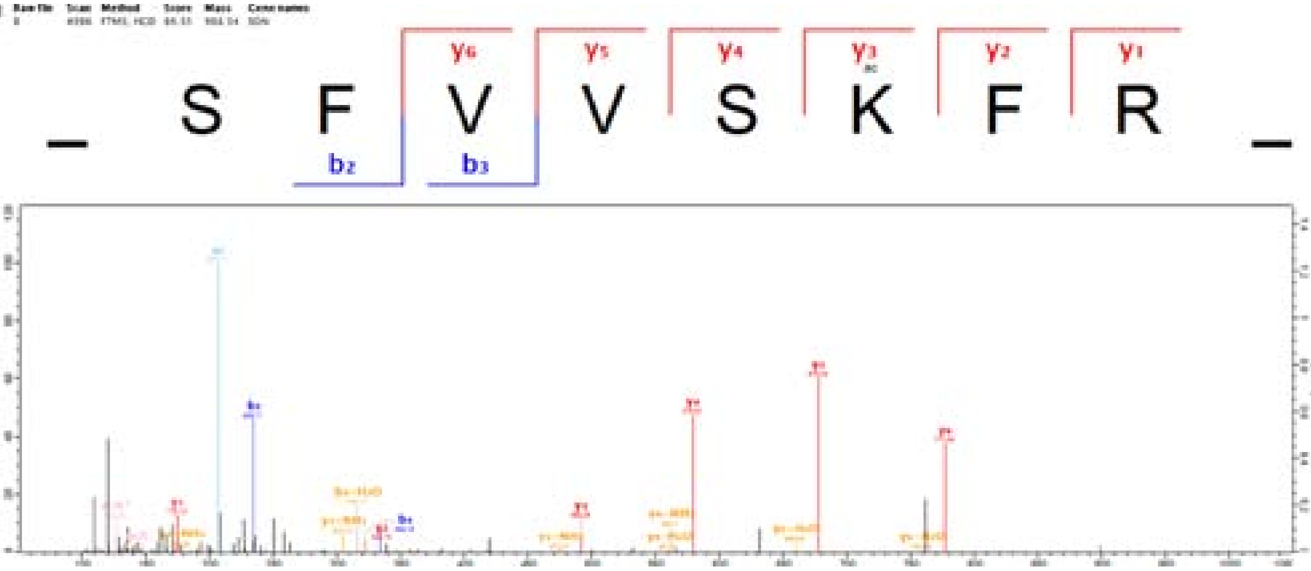
y6 y5 y4 y3 y2 y1
ac
_ S F V V S K F R _
b2 b3

J

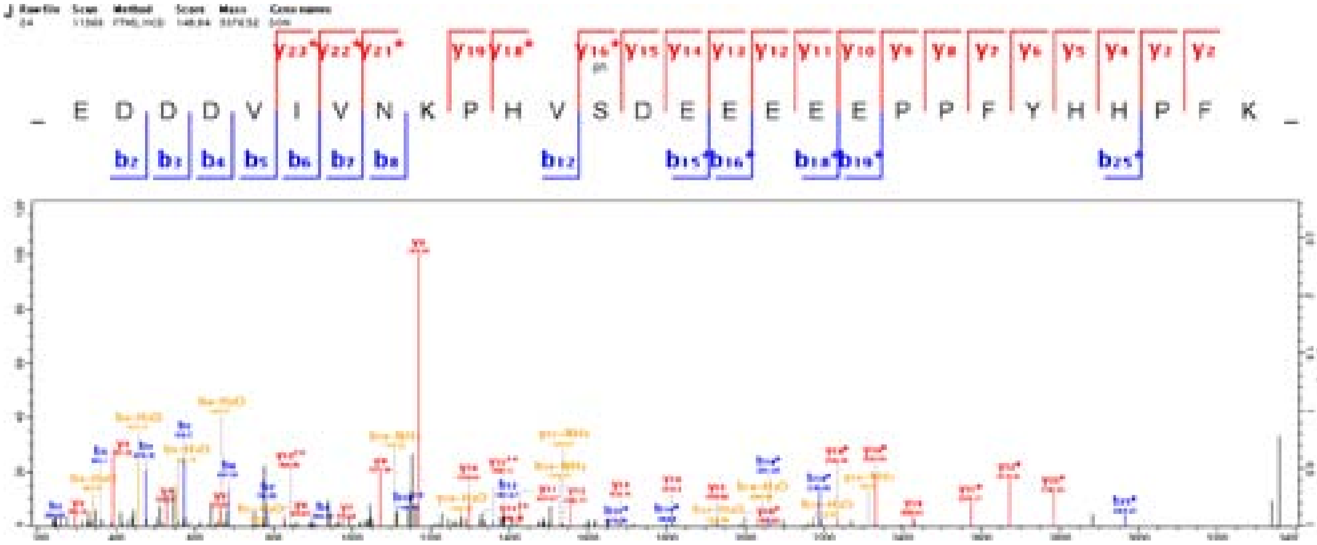
ph
_ E D D D V I V N K P H V S D E E E E E P P F Y H H P F K _
b2 b3 b4 b5 b6 b7 b8 b12

K

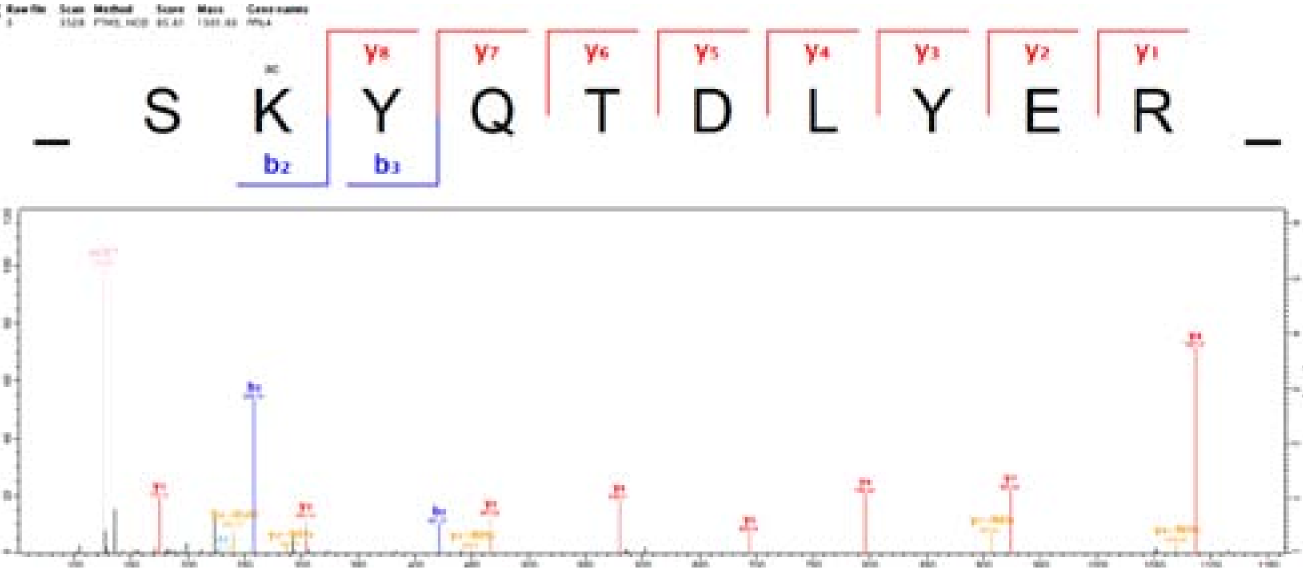
y8 y7 y6 y5 y4 y3 y2 y1
S K Y Q T D L Y E R
b2 b3

L

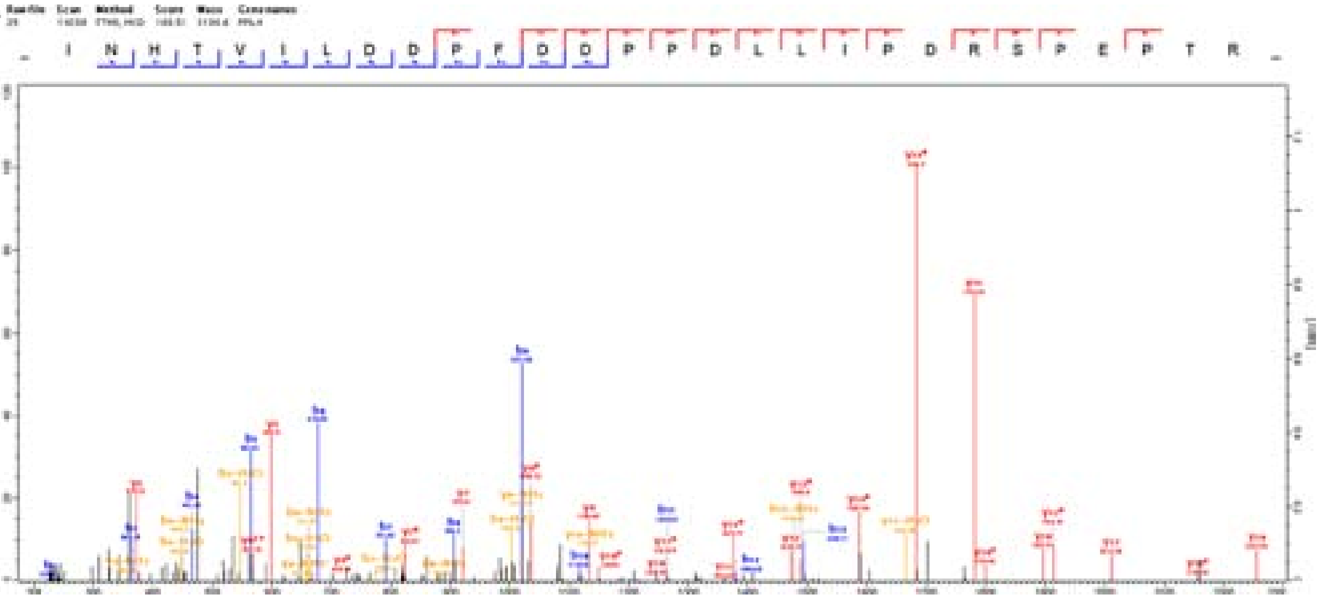
I N H T V I L D D P F D D P P D L L I P D R S P E P T R

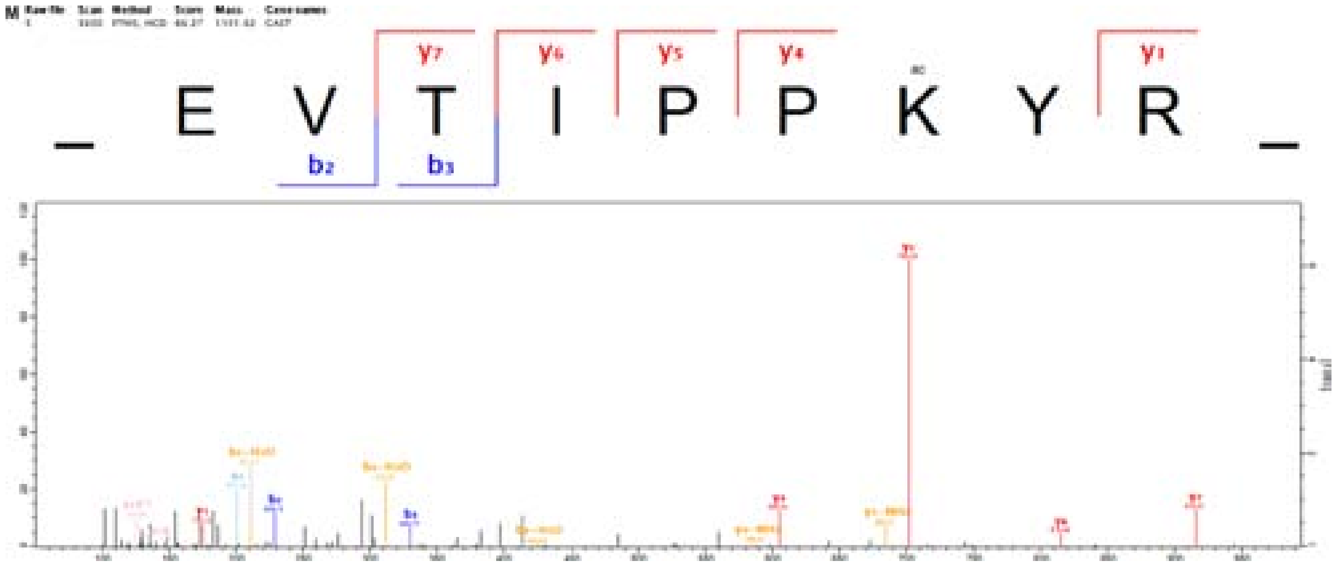

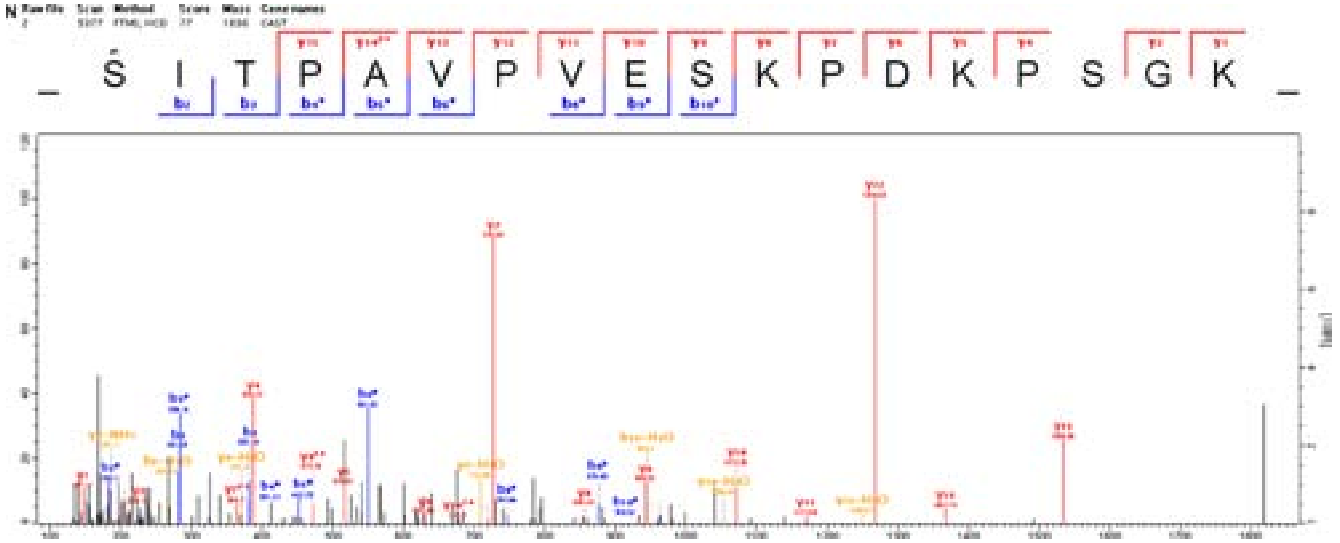

Supplementary Figure S1: MS/MS spectra of Q01105,O14617,Q9Y3Z3,Q6ZU65,P18583,Q8WUA2, and P20810 acetylation and phosphorylation. (**A**) Q01105 acetylation, (**B**) Q01105 phosphorylation, (**C**) O14617 acetylation, (**D**) O14617 phosphorylation, (**E**) Q9Y3Z3 acetylation, (**F**) Q9Y3Z3 phosphorylation, (**G**) Q6ZU65 acetylation, (**H**) Q6ZU65 phosphorylation, (**I**) P18583 acetylation, (**J**) P18583 phosphorylation, (**K**) Q8WUA2 acetylation, (**L**) Q8WUA2 phosphorylation, (**M**) P20810 acetylation, (**N**) P20810phosphorylation. These proteins were underwent both acetylation and phosphorylation.

Supplementary Table S1: Proteome profile changes analysis of HCT-8 and HCT-116 cells following FK228 treatment. See Supplementary_Table_S1

Supplementary Table S2: Alterations of phosphorylation profiles in FK228-treated HCT-8 and HCT-116 cells. See Supplementary_Table_S2

Supplementary Table S3: Protein-protein interaction networks associate with differentially phosphorylated proteins. See Supplementary_Table_S3

Supplementary Table S4: Acetylome profile alterations following FK228 treatment. See Supplementary_Table_S4

Supplementary Table S5: Protein-protein interaction network analysis of the differentially acetylated proteins. See Supplementary_Table_S5

Supplementary Table S6: Crosstalk between quantitated phosphorylation and the acetylome. See Supplementary_Table_S6
